# The Kinematics of Proal Chewing in Rats

**DOI:** 10.1093/iob/obae023

**Published:** 2024-07-26

**Authors:** E D McParland, J K Mitchell, J D Laurence-Chasen, L C Aspinwall, O Afolabi, K Takahashi, C F Ross, N J Gidmark

**Affiliations:** Department of Biology, Knox College, Galesburg, IL 61401, USA; Department of Ecology, Evolution and Organismal Biology, Brown University, Providence, RI 02912, USA; Department of Biology, Knox College, Galesburg, IL 61401, USA; Department of Organismal Biology & Anatomy, The University of Chicago, Chicago, IL 60637, USA; National Renewable Energy Laboratory, Golden, CO 80401, USA; Department of Biology, Knox College, Galesburg, IL 61401, USA; Department of Biology, Knox College, Galesburg, IL 61401, USA; College of Medicine, American University of Antigua, Osbourn, Antigua & Barbuda; Department of Organismal Biology & Anatomy, The University of Chicago, Chicago, IL 60637, USA; Department of Organismal Biology & Anatomy, The University of Chicago, Chicago, IL 60637, USA; Department of Biology, Knox College, Galesburg, IL 61401, USA; Department of Organismal Biology & Anatomy, The University of Chicago, Chicago, IL 60637, USA

## Abstract

Chewing kinematics are well-documented in several mammal species with fused mandibular symphyses, but relatively understudied in mammals with an unfused symphysis, despite the fact that more than half of extant Mammalia have an unfused mandibular symphysis. The Wistar brown rat (*Rattus norvegicus*) is widely used in human health research, including studies of mastication or neurological studies where mastication is the output behavior. These animals are known to have unfused mandibular symphyses and proal jaw (rostrocaudal) motion during occlusion, but the lack of high resolution, 3-dimensional analysis of rat chewing leaves the functional significance of symphyseal mobility unknown. We used biplanar fluoroscopy and the X-ray reconstruction of moving morphology workflow to quantify chewing kinematics in 3 brown rats, quantifying overall jaw kinematics, including motions about the temporomandibular joint and unfused mandibular symphysis. During occlusion, the teeth and the mandibular condyle translate almost exclusively anteriorly (proal) during occlusion, with little motion in any other degrees of freedom. At the symphysis, we observed minimal flexion throughout the chew cycle. Overall, there are fundamental differences in jaw kinematics between rats and other mammals and therefore rats are not an appropriate proxy for ancestral mammal jaw mechanics. Additionally, differences between humans and rat chewing kinematics must be considered when using rats as a clinical model for pathological feeding research.

## Introduction

Vertebrate chewing involves complex, 6-degree-of-freedom motion of the mandibles (Reilly et al. 2001; Ross et al. 2007; Gidmark et al. 2014; Schwarz et al. 2020). During occlusion (tooth-tooth contact), many mammals, including humans, chew with a lateral to medial power stroke, a movement thought to be associated with the evolution of tribosphenic molars (Crompton 1971, 1981; Hiiemäe 1984; Popowics and Herring 2006; Ross and Iriarte‐Diaz 2014). These lateral to medial jaw movements during the power stroke of mastication have been hypothesized to be facilitated by unfused mandibular symphyses which are argued to facilitate jaw roll (rotation about the hemimandible's long axis; Mills 1967; Kay and Hiiemäe 1974; Oron and Crompton 1985; Hylander and Johnson 1994; Lieberman and Crompton 2000; Reilly et al. 2001; Herring 2003; Bhullar et al. 2019, 2020; Zazhigin and Voyta 2019; Grossnickle 2020; Grossnickle et al. 2022; Stilson et al. 2023). Jaw yaw (rotation about a superoinferior axis) is an alternate strategy for “sided” chewing (food grinding on either the left or right side at any given time) using lateral to medial movements, and one that does not necessarily require an unfused symphysis (Kallen and Gans 1972; Gorniak 1977; Offermans and de Vree 1990; Menegaz et al. 2015; Grossnickle 2017; Iriarte-Diaz et al. 2017; Ross and Iriarte-Diaz 2019; Montuelle et al. 2020; Olson et al. 2021; Stilson 2021). These different motions suggest differential evolutionary paths for early mammal diversification of jaw motion (Patterson 1956; Crompton and Jenkins 1968; Gorniak 1977; Crompton and Parker 1978; Crompton 1981; Oron and Crompton 1985; Offermans and de Vree 1990; Druzinsky 1995; Mao et al. 2020; Schultz 2020) and complex molar morphology (Patterson 1956; Grossnickle 2017; Bhullar et al. 2019, 2020; Mao et al. 2020; Schultz 2020).

**Fig. 1:**
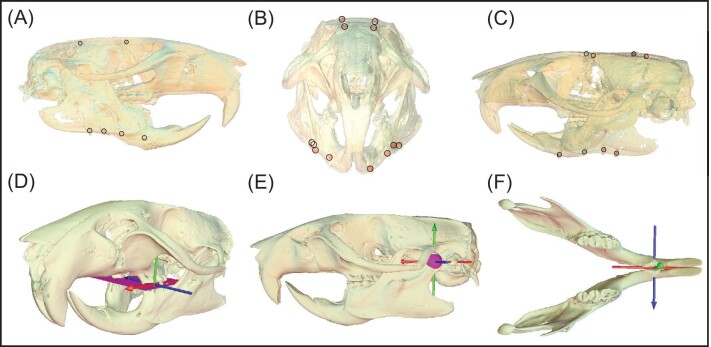
Marker constellation location and coordinate system orientation. Four tantalum beads were surgically implanted into each of the following bones of 3 rats (*Rattus norvegicus*): cranium, left hemimandible, and right hemimandible seen from lateral right (A), anterior (B), and lateral left (C) views. An ACS was aligned (see methods for information on alignment) using the upper tooth row occlusal plane (created in Maya; plane) and positioned in the centroid of the second lower molar (D). Lower molar tooth translations were measured relative to the cranium via this ACS. The temporomandibular JCS was positioned at the centroid of the mandibular condyle (E; sphere; following Brainerd et al. 2010 and Menegaz et al. 2015). Symphysis coordinate system was placed at the centroid of the symphysis with ACS axes attached to left and right hemimandibles (F). Note that the depicted posture in (D–F) represents the “zero” position of the joint coordinate systems (i.e. fixed and mobile axes overlapping).

However, not all mammals chew with medial jaw movements; anteriorly-directed (proal) jaw movements during the power stroke evolved early on in the therian lineage and are seen in many extant taxa (Gorniak 1977; Herring 1993; Landry 1954; Wall 1999; Luo et al. 2001; Williams et al. 2007; Davis 2011; Luo et al. 2017; Olson et al. 2021; Grossnickle et al. 2022). In particular, Rodentia, a highly diverse clade of mammals that includes rats and mice, have diverse chewing modes, with some exhibiting proal movement during the power stroke (Atchley 1993; Druzinsky 1995; Monteiro et al. 2005; Michaux et al. 2007; Hautier et al. 2011, 2012). These motions are facilitated by skeletal morphology of the temporomandibular joint (TMJ) in this lineage. In humans, the articular eminence (a protrusion of the temporal bone anterior to the joint fossa) restricts anterior condylar translation. In rats, the TMJ is shallow and lacks an articular eminence, allowing for relatively unrestricted anterior translation of the condyle (Fig. 1; Weijs 1975; Herring 2003; Porto et al. 2010; Orset et al. 2014). Further flexibility in mandibular motion is permitted by a ligamentous mandibular symphysis, which permits semi-independent movements of the left and right lower jaw bones (“hemimandibles”; Oron and Crompton 1985; Lieberman and Crompton 2000; Herring 2003; Porto et al. 2010; Orset et al. 2014). This is in contrast with the fused mandibular symphysis of many other mammals (Hiiemäe and Houston 1971; Weijs 1975; Beecher 1979; Hylander et al. 2000; Lieberman and Crompton 2000; Porto et al. 2010). Symphyseal fusion increases strength, enabling force transfer from the balancing (non-chewing) side to the bite point (Hylander et al. 2000), but it eliminates the independence of hemimandibular motion thought to facilitate precise occlusion during mastication in therians (Crompton and Hiiemäe 1970; Oron and Crompton 1985). The presence of unfused mandibular symphyses in many vertebrates that lack precise occlusion has been suggested to confer other functional advantages (Gorniak 1977; Beecher 1979; Lauder 1980; Scapino 1981; Druzinsky 1995; Lieberman and Crompton 2000; Ravosa and Hogue 2004; Gintof et al. 2010; Gidmark et al. 2014; Laurence-Chasen et al. 2019). Despite this diversity of occlusal mode, kinematic studies of mammals with unfused mandibular symphyses have focused on taxa with lateral to medial movements of the teeth during the power stroke (Davis 2014; Bhullar et al. 2019; Stilson et al. 2023): less attention has been paid to species employing posterior to anterior (P-to-A, proal) or anterior to posterior (A-to-P, palinal) jaw movements during the power stroke.

Rats provide an opportunity to investigate the range of jaw movements facilitated by the combination of an unconstrained TMJ and semi-independent hemimandibles in taxa in which proal translation of the molars is the predominant jaw movement during the power stroke of chewing (Simpson 1936; Hiiemäe and Ardran 1968). Though previous studies have examined the kinematics of chewing in rats (Hiiemäe 1971a,b; Weijs 1975; Byrd 1988), a thorough, quantitative, 6-degree-of-freedom kinematic study of rodent chewing has not yet been conducted. Most characterizations of jaw motions in rodents are based on tooth wear patterns and muscle structure and activity (Hiiemäe 1971a,b; Hiiemäe and Houston 1971; Teaford and Byrd 1989; Offermans and deVree 1993; Druzinsky 2010; Cox and Jeffery 2015; Ungar and Sues 2019; Williams 2019; Grossnickle et al. 2022), but without evaluating whether these morphological and muscle activity patterns can accurately predict jaw kinematics.

The ready availability and inducible pathologies of the TMJ in rats and mice makes them common model organisms for studies of TMJ disorders (TMDs; Roveroni et al. 2001; Porto et al. 2010; Sperry et al. 2019). Studies of rodents have yielded significant insights into the biomechanical and neurological implications of TMD (Takeda et al. 2006; Ellenbroek and Youn 2016; Yotsuya et al. 2019, 2020; Moriuchi et al. 2019;Reed et al. 2019, 2021; Qiao et al. 2022). However, despite their importance as clinical models, rigorous quantification of 6-degree-of-freedom hemimandibular motions during chewing in healthy rats have not previously been reported.

Here, we quantify 6-degree-of-freedom hemimandibular motion in healthy *Rattus norvegicus* (Berkenhout 1769) using biplanar videofluoroscopy and the X-ray reconstruction of moving morphology workflow (XROMM; Brainerd et al. 2010; Gatesy et al. 2010) with the goals of: (1) describing overall kinematics of the jaw during chewing; (2) understanding the implications of those kinematics for molar translation during occlusion; and (3) quantifying the magnitude of symphyseal flexion.

## Materials and methods

All husbandry and experimental procedures followed University of Chicago IACUC Protocol 72440. Under isoflurane anesthesia, radio-opaque tantalum sphere markers (0.8–1.0 mm, X-medics, Frederiksberg, Denmark) were surgically implanted into bones of 3 Wistar brown rats (*Rattus norvegicus*; Berkenhout 1769) using manual drill bits and forceps. Four markers were placed in the cranium, and left and right hemimandibles of each individual (Fig. 1A–C), with the exception of rat B which had only 3 right hemimandible markers successfully implanted. After surgery, the rats were allowed to heal and recover for a minimum of 1 week before recording feeding trials.

Standard marker-based XROMM protocols (Brainerd et al. 2010; Gatesy et al. 2010) were followed to generate 3D reconstructions of hemimandibular and cranial motions during feeding. In brief, biplanar X-ray videos (parameters: 250 frames/s; 85 kV; 32 mA) were recorded while the rats chewed pellet food. Image undistortion, 3D camera calibration, marker tracking, and rigid body transformation reconstruction were performed with XMALab (version 1.5.5; Knörlein et al. 2016). Trials and frames with low contrast quality were removed from the dataset because they would lead to high reprojection error, rigid body transform error, or grid correction error. Rats were CT scanned before and after marker implantation using a Vimago^TM^ CT scanner (Epica International Inc., Duncan, SC, USA) in the University of Chicago Animal Resources Center. Image stacks were segmented using 3D Slicer (v4.11, National Institutes of Health, Bethesda, MD, USA) to generate digital mesh models of each individual skull bone. Computed rigid body transformation data (filtered natively in XMALab using a low-pass Butterworth filter between 20 and 5 Hz; Fig. S1) from XMALab were applied to the bone models to reconstruct and visualize precise *in vivo* motions of chewing in Autodesk Maya (v2020, Autodesk, Inc., San Rafael, CA, USA).

A cranial anatomical coordinate system (ACS) was aligned by fitting a plane to the cusps of the upper tooth row (Fig. 1D; Brainerd et al. 2010; Menegaz et al. 2015). Anterior and lateral axes were defined by this plane using the long axis of the cranium as positive *X*. Lateral was defined as orthogonal to *X* (positive to the animal's right) and constrained to the tooth cusp plane. The *Y* axis of the ACS was defined orthogonal to the *X* and *Z* axes with superior being positive. Six-degree-of-freedom measurements of both the temporomandibular (Fig. 1E) and symphyseal (Fig. 1F) joints were obtained through 2 separate joint coordinate systems (JCS; Grood and Suntay 1983; Brainerd et al. 2010). First, the TMJ JCS was positioned at the centroid of the mandibular condyle, and comprised the cranial ACS as well as a mandibular ACS (with identical alignment to the cranial ACS). In JCS calculation, the cranial ACS was used as the fixed (proximal) element, and the mandibular ACS as the mobile (distal) element. The “zero” pose of the JCS was selected as a centric occlusion of the midpoint of a representative chew in an *in vivo* posture. Second, the symphyseal JCS was positioned at the centroid of the mandibular symphysis, and comprised 2 ACSs aligned with the tooth cusp plane described for the cranial ACS, with the left hemimandible as the fixed element and the right hemimandible as the mobile element (Fig. 1F). Due to the hierarchical nature of JCS function in the XROMM workflow (following Grood and Suntay 1983), the *Z* (lateral) axis is fixed to the proximal bone and the *X* (anterior) axis is fixed to the distal bone; the *Y* axis is computed as orthogonal to the other 2 axes. Note that assigning the left versus right hemimandible as the fixed element only impacts the polarity of the JCS rotation values, and not their magnitude. We measured translations of the second molar by measuring the motion of a digital locator on its occlusal surface relative to the cranial ACS, after translating the ACS to the center of the maxillary occlusal surface (Fig. 1D). Data were exported from Maya as CSV files using scripts in the XROMM MayaTools shelf (developed by Dave Baier; https://bitbucket.org/xromm/xromm_mayatools/src/master/).

**Fig. 2:**
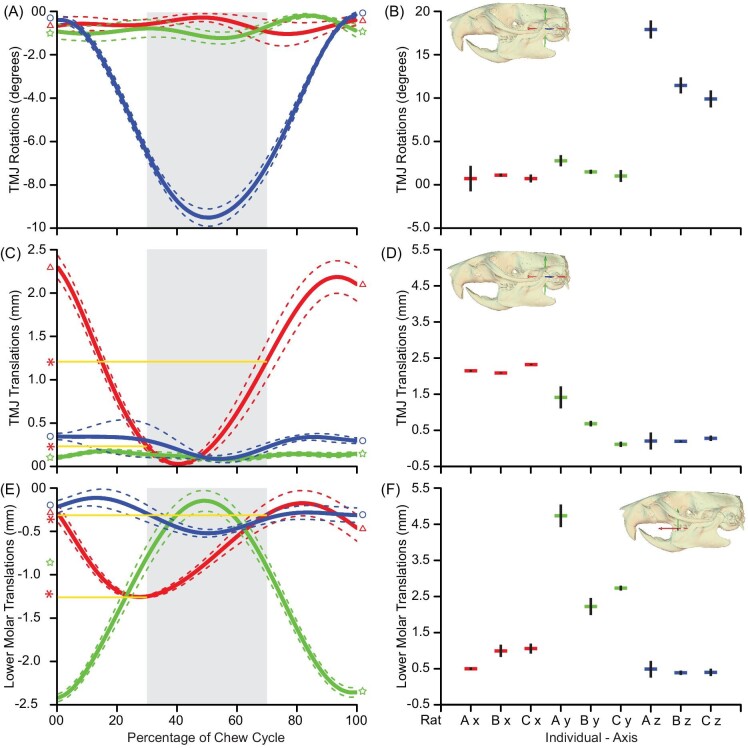
Chew summary data. Chew cycles were cropped and spline-interpolated to summarize 6 degrees of motion throughout the chew cycle. Measurements were extracted from coordinate systems at the TMJ for rotations (A–B) and translations (C–D) of the mandible and translations (E–F) of the lower second molar. Panels A, D, and E are summarized from an individual rat. Panels B, D, and F are summary data of the absolute amplitude of motions across 3 individuals. Gray shaded area = occlusal phase, dashed lines = 95% confidence intervals, A–P translation during occlusion = yellow lines/red asterisks. Coordinate system axes: anteroposterior (*X*; red; triangle trace); superoinferior (*Y*; green; star trace); and transverse (=mediolateral; right; *Z*; blue; circle).

Our final dataset only included chew cycles during tooth occlusion (instead of manipulation, transport, gathering, swallowing, or other non-feeding behaviors). It totaled to 38 chews was quantified (rat A: *n* = 13; rat B: *n* = 7, and rat C: *n* = 18). Visual inspection of reconstructions showed that the occlusal phase occupied approximately the middle 40% of the chew cycle. Each cycle was spline-interpolated to 100 data points in R studio (Version 4.2.0; Fig. 2 traces Rat C). Cycles were compiled and aligned for statistical purposes on a rat-by-rat basis. This custom code is available at https://bitbucket.org/ratfeedingxromm/workspace/projects/KPC.

Three additional cadaveric rat heads were marked with the same surgical approach to investigate precision of 6DOF measurements. After marking, heads were frozen, vacuum-sealed and waved in the X-ray video space to quantify the range of error inherent in the XROMM workflow (reported in Table 1). With no movement of the frozen jaws relative to the cranium, error was only attributed to workflow error (Menegaz et al. 2015). Workflow versus between-cycle confidence intervals are compared in Table 1.

**Table 1:** Summary data of absolute amplitude of 6DOF motions and error across 3 individuals’ chew cycles and workflow error.

	Condylar Rx (jaw roll; degrees)	Condylar Ry (jaw yaw; degrees)	Condylar Rz (jaw pitch; degrees)	Condylar Tx (mm)	Condylar Ty (mm)	Condylar Tz (mm)	Lower Molar Tx (mm)	Lower Molar Ty (mm)	Lower Molar Tz (mm)	Symphyseal Rx (degrees)	Symphyseal Ry (degrees)	Symphyseal Rz (degrees)	Symphyseal Tx (mm)	Symphyseal Ty (mm)	Symphyseal Tz (mm)
Rat A: absolute magnitude of motion (Average Between-cycle CI)	0.80 (1.48)	2.86 (0.65)	18.10 (1.04)	2.13 (0.02)	1.40 (0.31)	0.19 (0.24)	0.50 (0.04)	4.73 (0.31)	0.49 (0.23)	1.10 (1.03)	1.13 (0.50)	1.55 (0.55	0.18 (0.05)	0.62 (0.24)	0.41 (0.12)
Rat B: absolute magnitude of motion (Average Between-cycle CI)	1.19 (0.17)	1.63 (0.24)	11.60 (0.93)	2.08 (0.01)	0.37 (0.01)	0.18 (0.02)	0.99 (0.01)	2.51 (0.21)	0.39 (0.06)	2.09 (1.23)	0.60 (0.32)	0.63 (0.54)	0.04 (0.02)	0.36 (0.29)	0.20 (0.08)
Rat C: absolute magnitude of motion (Average Between-cycle CI)	0.81 (0.46)	1.11 (0.69)	10.00 (0.99)	2.31 (0.1)	0.10 (0.07)	0.27 (0.08)	1.06 (0.14)	2.22 (0.27)	0.40 (0.10)	0.99 (1.11)	1.50 (0.60)	0.82 (0.32)	0.04 (0.04)	0.06 (0.03)	0.08 (0.07)
Workflow CI	0.07	0.017	0.021	0.00097	0.0012	0.0016	–	–	–	-	-	-	-	-	-

Absolute amplitude of rotational and translational motions of the TMJ, translational measurements of the lower molar, and rotational and translational measurements of the mandibular symphysis. Average between-cycle 95% confidence intervals were calculated on an individual basis. Cadaveric workflow precision 95% confidence intervals are also reported. Coordinate system axes: anteroposterior (*X*; red); superoinferior (*Y*; green); and transverse (=mediolateral; right; *Z*; blue). *R* = rotations, *T* = translations.

## Results

### General observations of feeding behavior

During normal chewing (Fig. 2; Fig. S1), mandibular depression opens the mouth and elevation closes it. Though this pitching (Rz; blue trace in Fig. 2A) is key to chewing, other important movements also occur. During elevation, lower molars translate along a superoinferior axis (Ty; green trace in Fig. 2C). Once the TMJ maximally pitches (Rz) into the occlusal phase (Fig. 2A), the lower tooth row undergoes proal movement: the lower molars translate along the uppers in a posterior-to-anterior direction (Tx; red trace in Fig. 2E). This is the only translation during occlusion. Condylar pitching (Rz; Fig. 2A) produces lower molar superoinferior translations (Ty; Fig. 2E), but also produces molar anteroposterior translations throughout the chew cycle (Tx; red trace in Fig. 2B).

### 6DOF condylar motions at the TMJ

We observed a range across individuals (referred to only as “range” hereafter) of 10.0°–18.1° (Fig. 2B; Table 1) of pitching from peak opening to complete occlusion (Rz; Fig. 2A, Table 1). The magnitude of pitching was greater during food gathering cycles. Roll (Rx; red trace in Fig. 2A) is inconsistent and minimal at ranging 0.8°–1.2° (Fig. 2B; Table 1) during chew cycles (Fig. 2A). Similarly, the mandible yaws minimally (Ry; green trace in Fig. 2A), only ranging from 1.1° to 2.9° at the TMJ (Fig. 2B; Table 1) throughout the chew cycles (Fig. 2A). Individual absolute amplitude kinematics were similar overall (Fig. 2B; Table 1). Between-cycle variation and workflow error for all kinematic variables are reported in Table 1.

Superoinferior (Ty; green trace in Fig. 2C) and mediolateral (Tz; blue trace in Fig. 2C) translations at the TMJ were minimal throughout the chew cycle. Superoinferior (Ty; 0.1–1.4 mm; Fig. 2D; Table 1) and mediolateral (Tz; 0.2–0.3 mm; Fig. 2D; Table 1) translations were inconsistent and negligible throughout the chew cycle with less than 0.1 mm during occlusion (Fig. 2C; Table 1). The largest translation at the TMJ was anterior (Tx; red trace in Fig 2C), with a range in magnitude of 2.1–2.3 mm (Fig. 2D; Table 1), with 1.2 mm of anterior translation occurring during the occlusal phase (Fig. 2C). Most of this anteriorly directed condylar translation happened during occlusion (Fig 2C; see yellow horizontal line and red asterisk). Individual translational absolute amplitude kinematics were similar overall (Fig 2D; Table 1).

### Translations of the lower tooth row

**Fig. 3:**
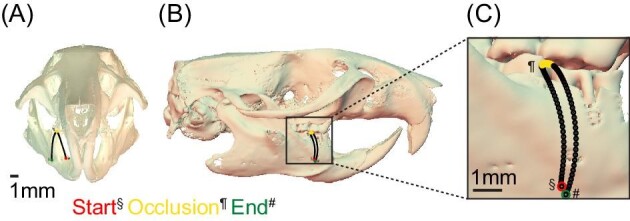
Hemimandibles move anteriorly during occlusion. Anterior (A) and lateral (B and C) visualization of tooth movement illustrates mediolateral translation during open and close, but only anteriorly-directed movement during occlusion. Each data point (black) represents one % of the chew cycle. Red (§) = start, mouth open, yellow (¶) = occlusion, and green (#) = end, mouth open.

Lower molar posterior-to-anterior translation (Tx; red trace in Fig. 2E; Fig. 3) mirrored the condyle, ranging 0.5–1.1 mm (Fig. 2F; Table 1), nearly reaching its absolute maximum during occlusion (Fig. 2E; see yellow horizontal line and red asterisk). Molars translated superoinferiorly (Ty; green trace in Fig. 2E) ranging 2.2–2.5 mm (Fig. 2; Table 1) during mouth opening/closing but minimally during peak occlusion (Fig. 2E). We observed minimal (>0.25 mm; Fig. 2F; Table 1) and inconsistent mediolateral translations (Tz; blue trace in Fig. 2E) throughout chew cycles and during occlusal phases (Fig. 2E). Individual translation absolute amplitude kinematics were similar overall (Fig. 2F; Table 1).

### 6DOF symphyseal joint motions

**Fig. 4:**
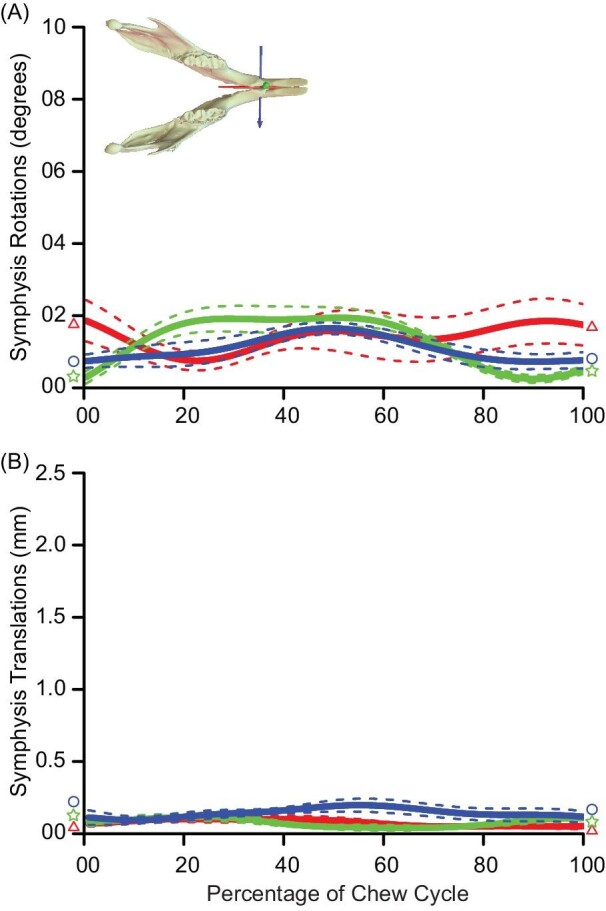
Summary kinematics of the mandibular symphysis. Rotational (A) and translational (B) data were cropped and spline-interpolated to summarize 6 degrees of motion to summarize the whole chew cycle. *Y*-axis scaling is based on rotations/translations of the TMJ (Fig. 2A and C). Coordinate system axes: anteroposterior (*X*; red; triangle trace); superoinferior (*Y*; green; star trace); and transverse (=mediolateral; right; *Z*; blue).

Measurement of 6-degrees-of-freedom kinematics at the mandibular symphysis revealed minimal motion between left and right hemimandibles throughout chew cycles. Absolute magnitudes of rotations about all 3 axes averaged 1.15° (Fig. 4A; Table 1); absolute magnitudes of translations along these degrees of freedom averaged 0.2 mm (Fig. 4B; Table 1).

## Discussion

### Exclusive proal jaw motions during occlusion

Chewing kinematics and morphology-based studies on mammals have traditionally emphasized species that use primarily lateral-to-medial tooth movements during occlusion (Herring and Scapino 1973; Herring 1976; Dantuma and Weijs 1980; Brehnan et al. 1981; Gorniak 1985; Williams et al. 2011; Menegaz et al. 2015; Iriarte-Diaz et al. 2017), and mostly (but not entirely, Bhullar et al. 2019; Stilson et al. 2023) on species with fused mandibular symphyses. Our data extends these studies to a species with an unfused symphysis, and which has long been known to employ a primarily proal power stroke (Weijs 1975). Our 3-dimensional, 6-degree-of-freedom analysis reveals that rats employ almost exclusively proal kinematics during occlusion (Fig. 3), consistent with anatomical inferences made in previous rodent studies (Hiiemäe and Ardran 1968; Hiiemäe 1971a,b; Hiiemäe and Houston 1971; Weijs 1975; Byrd 1981, 1988; Druzinsky 1995; Reilly et al. 2001; Cox et al. 2012; Ungar and Sues 2019; Williams 2019). Anterior translations of molars and condyles are nearly identical, suggesting that anterior molar translation during occlusion is driven by mandibular translation (Fig. 2), not other mandibular motions (e.g., roll, yaw). In rats, anterior translation of the condyle continues not only during occlusion, but also during mouth opening, as in other species (Gorniak 1977; Brehnan et al. 1981; Druzinsky 1995; Williams et al. 2011; Henderson et al. 2014; Menegaz et al. 2015; Iriarte-Diaz et al. 2017; Stover et al. 2017). Jaw yaw and roll were minimal, and would not result in anterior translation of the molars (Grossnickle 2017, 2020; Bhullar et al. 2019). The anterior translation of the condyle during occlusion suggests that the loading regime of the TMJ is likely different from that seen in other species, including humans, that do not use proal chewing kinematics (Koolstra and Van Eijden 1995; Herring and Liu 2001; Tuijt et al. 2010).

### Anatomical implications for jaw kinematics

Exclusively anterior translations during occlusion are reflected in craniofacial anatomy. Rat TMJs are anteroposteriorly elongate, and with lateral eminences that likely constrain mandibular translation to an anterior direction during occlusion (Hiiemäe and Houston 1971; Walker and Homberger 1997). This morphology allows translation along the fossa that would be anatomically impossible with restricted anterior and/or posterior eminences (Osborn 1989; Yatabe et al. 1997; Mesnard et al. 2012). In rats, despite the unfused mandibular symphysis, the left and right hemimandibles are loaded equally, with bilaterally symmetric adductor muscle firing patterns (Weijs and Dantuma 1975). Jaw elevator muscles in rats have large components oriented parallel to the long axis of the mandible (Hiiemäe and Houston 1971; Cox and Jeffery 2015; Hiiemäe 1971b) and are active during proal grinding (Easton and Carison 1990).

### Evolutionary implications of an unfused symphysis for jaw kinematics

Extant and fossil mammals have diverse dental morphologies and masticatory motion patterns. Early mammaliaforms are thought to have used either predominantly orthal or palinal (posteriorly directed) motions during mastication (Crompton and Hotton 1967; Angielczyk 2004; Schultz et al. 2014; Luo et al. 2015; Martin et al. 2020; Grossnickle et al. 2022). Diversification of the feeding apparatus among therians is hypothesized to be a significant factor in the evolution of this group, with an emphasis on tribosphenic molars and a dorsomedial movement of the lower molars during the power stroke, facilitated by the evolution of an unfused symphysis. Mandibular roll is an important movement for occlusion of tribosphenic molars and is facilitated by an unfused symphysis (Kay and Hiiemäe 1974; Bhullar et al. 2019, 2020; Grossnickle 2020).

The fusion of hemimandibles at the symphysis has arisen multiple times among crown mammals and its functional significance is well studied (e.g., Beecher 1979; Scapino 1981; Hylander 1979, 1984; Druzinsky 1995; Lieberman and Crompton 2000; Ravosa and Hogue 2004; Williams et al. 2008). Extant mammals with an unfused mandibular symphysis have 2 separate hemimandibles that are thought to move semi-independently from one another (Kay and Hiiemäe 1974; Oron and Crompton 1985; Hylander and Johnson 1994; Lieberman and Crompton 2000; Bhullar et al. 2019; Zazhigin and Voyta 2019). These species are commonly used for *in vivo* studies that make inferences about jaw kinematics of early mammals (Bhullar et al. 2019, 2020; Stilson 2021; Stilson et al. 2023). Bhullar and colleagues (2019) revealed that *Monodelphis* employ their flexible symphysis for sided teeth grinding through rolling (rather than yawing) of the hemimandibles and argue these motions as a model of ancestral tribosphenic therian kinematics. Previous work from Weijs (1975) revealed exclusive bilateral chewing in rats (and therefore lacking a working/balancing side) and hypothesized that a movable symphysis might permit adjustment of loading force on either side of the jaw. Although the current study also observed bilateral chewing, there were minimal movements in any degree of freedom in the symphyseal joint (Fig. 4). Our data reveal that although rats also have unfused symphyses, they use minimal roll, yaw, mediolateral translation, or symphyseal flexion, instead emphasizing proal jaw movements during occlusion. Thus, rodent chewing is not an appropriate proxy or functional analogue to mammals, like humans, with tribosphenic teeth, lateral-medial tooth movements during occlusion, or early ancestral mammal jaw kinematics.

### Using rats as a clinical model organism for human TMD Research

Rats and mice are common model organisms in clinical studies of human temporomandibular joint disorders (TMDs) because of their ready availability and associated genetic manipulation technology (Porto et al. 2010; Sangani et al. 2015; Moriuchi et al. 2019; Yotsuya et al. 2019; Sun et al. 2020). However, this study demonstrates the basic pattern of TMJ motion that characterizes rat chewing is fundamentally different than seen in humans. This calls into question the extent to which rodent models of TMD are relevant for understanding the biomechanical underpinnings of human TMD. Other model organisms (i.e., monkeys and pigs) are anatomically and kinematically more similar to humans, however, they are less accessible and less manipulable (Bermejo et al. 1993; Herring et al. 2002; Herring 2003; Abraha et al. 2022). Therefore, clinical studies of TMD should consider the discrepancies between rodent and human TMJ morphology and kinematics during biomechanical analysis.

## Conclusion

Mastication in rats is characterized by whole-jaw proal motions. While these kinematics have been qualitatively described in the literature, we reveal the exclusivity of proal motions in left and right hemimandibles. Unlike other stem mammals with unfused mandibular symphyses, we observed negligible roll and yaw of the jaws, making rodent mastication distinct from most early mammals. Because of these novel traits in masticatory mode and musculoskeletal morphology, the use of rats as a model for human jaw kinematic clinical research needs to be conducted critically.

## Supplementary Material

obae023_Supplemental_File

## References

[bib1] Abraha HM , Iriarte-Diaz J, Reid RR, Ross CF, Panagiotopoulou O. 2022. Fracture fixation technique and chewing side impact jaw mechanics in mandible fracture repair. JBMR Plus 6:e10559. 35079674 10.1002/jbm4.10559 PMC8770999

[bib2] Angielczyk KD. 2004. Phylogenetic evidence for and implications of a dual origin of propaliny in anomodont therapsids (Synapsida). Paleobiology 30:268–96.

[bib3] Atchley WR. 1993. Genetic and developmental aspects of variability in the mammalian mandible. Skull 1:207–47.

[bib4] Beecher RM. 1979. Functional significance of the mandibular symphysis. J Morphol 159:117–30. 423251 10.1002/jmor.1051590109

[bib4a] Berkenhout J . 1769. Outlines of the natural history of Great Britain and Ireland. London: P. Elmsly.

[bib5] Bermejo A , González O, González JM. 1993. The pig as an animal model for experimentation on the temporomandibular articular complex. *Oral Surgery, Oral Medicine*. Oral Pathol 75:18–23. 10.1016/0030-4220(93)90399-o 8419867

[bib6] Bhullar BAS , Manafzadeh AR, Miyamae JA, Hoffman EA, Brainerd EL, Musinsky C, Crompton AW. 2019. Rolling of the jaw is essential for mammalian chewing and tribosphenic molar function. Nature 566:528–32. 30760927 10.1038/s41586-019-0940-x

[bib7] Bhullar BAS , Manafzadeh AR, Miyamae JA, Hoffman EA, Brainerd EL, Musinsky C, Crompton AW. 2020. Reply to: jaw roll and jaw yaw in early mammals. Nature 582:E9–E12. 32555494 10.1038/s41586-020-2364-z

[bib8] Brainerd EL , Baier DB, Gatesy SM, Hedrick TL, Metzger KA, Gilbert SL, Crisco JJ 2010. X-ray reconstruction of moving morphology (XROMM): precision, accuracy and applications in comparative biomechanics research. J Exp Zool A: Ecol Genet Physiol 313:262–79. 20095029 10.1002/jez.589

[bib9] Brehnan K , Boyd RL, Laskin J, Gibbs CH, Mahan P. 1981. Direct measurement of loads at the temporomandibular joint in Macaca arctoides. J Dent Res 60:1820–4. 6944346 10.1177/00220345810600101501

[bib10] Byrd KE. 1981. Mandibular movement and muscle activity during mastication in the guinea pig (Cavia porcellus). J Morphol 170:147–69. 7299825 10.1002/jmor.1051700203

[bib11] Byrd KE. 1988. Opto-electronic analyses of masticatory mandibular movements and velocities in the rat. Arch Oral Biol 33:209–15. 3178540 10.1016/0003-9969(88)90047-7

[bib12] Cox PG , Jeffery N. 2015. The muscles of mastication in rodents and the function of the medial pterygoid. Evol Rodents 5:350–72.

[bib13] Cox PGR , E. J, Fagan MJ, Herrel A, Pataky TC, Jeffery N. 2012. Functional evolution of the feeding system in rodents. PLoS One 7:e36299. 22558427 10.1371/journal.pone.0036299 PMC3338682

[bib14] Crompton AW. 1971. The origin of the tribosphenic molar. Early mammals 50:65–87.

[bib15] Crompton AW. 1981. The origin of mammalian occlusion. In: Barrer HG, editor. Orthodontics. Philadelphia, PA: University of Pennsylvania Press, p. 3–18.

[bib16] Crompton AW , Hiiemäe K. 1970. Molar occlusion and mandibular movements during occlusion in the American opossum, *Didelphis marsupialis* L. Zool J Linn Soc 49:21–47.

[bib17] Crompton AW , Hotton N. III 1967. Functional morphology of the masticatory apparatus of two dicynodonts (Reptilia, Therapsida). Postilla 109:1–51.

[bib18] Crompton AW , Jenkins FA. 1968. Molar occlusion in Late Triassic mammals. Biol Rev 43:427–58. 4886687 10.1111/j.1469-185x.1968.tb00966.x

[bib19] Crompton AW , Parker P. 1978. Evolution of the mammalian masticatory apparatus: the fossil record shows how mammals evolved both complex chewing mechanisms and an effective middle ear, two structures that distinguish them from reptiles. Am Sci 66:192–201. 646211

[bib20] Dantuma R , Weijs WA. 1980. Functional anatomy of the masticatory apparatus in the rabbit (Oryctolagus cuniculus L.). Netherlands J Zool 31:99–147.

[bib21] Davis BM. 2011. Evolution of the tribosphenic molar pattern in early mammals, with comments on the “dual-origin” hypothesis. J Mamm Evol 18:227–44.

[bib22] Davis JS. 2014. Functional morphology of mastication in musteloid carnivorans. PhD Thesis. Ohio University, Athens, OH.

[bib23] Druzinsky RE. 1995. Incisal biting in the mountain beaver (*Aplodontia rufa*) and woodchuck (*Marmota monax*). J Morphol 226:79–101. 7473765 10.1002/jmor.1052260106

[bib24] Druzinsky RE. 2010. Functional anatomy of incisal biting in *Aplodontia rufa* and sciuromorph rodents–Part 1: masticatory muscles, skull shape and digging. Cells Tissues Organs 191:510–22. 20160428 10.1159/000284931 PMC2883844

[bib25] Easton JW , Carison DS. 1990. Adaptation of the lateral pterygoid and superficial masseter muscles to mandibular protrusion in the rat. Am J Orthod Dentofacial Orthop 97:149–58. 2301301 10.1016/0889-5406(90)70088-t

[bib26] Ellenbroek B , Youn J. 2016. Rodent models in neuroscience research: is it a rat race? Dis Model Mech 9:1079–87. 27736744 10.1242/dmm.026120 PMC5087838

[bib27] Gatesy SM , Baier DB, Jenkins FA, Dial KP. 2010. Scientific rotoscoping: a morphology-based method of 3-D motion analysis and visualization. J Exp Zool A: Ecol Genet and Physiol 313:244–61. 20084664 10.1002/jez.588

[bib28] Gidmark NJ , Tarrant JC, Brainerd EL. 2014. Convergence in morphology and masticatory function between the pharyngeal jaws of grass carp, Ctenopharyngodon idella, and oral jaws of amniote herbivores. J Exp Biol 217:1925–32. 24577451 10.1242/jeb.096248

[bib29] Gintof C , Konow N, Ross CF, Sanford CP. 2010. Rhythmic chewing with oral jaws in teleost fishes: a comparison with amniotes. J Exp Biol 213:1868–75. 20472774 10.1242/jeb.041012

[bib30] Gorniak GC. 1977. Feeding in golden hamsters, *Mesocricetus auratus*. J Morphol 154:427–58. 592408 10.1002/jmor.1051540305

[bib31] Gorniak GC. 1985. Trends in the actions of mammalian masticatory muscles. Am Zool 25:331–8.

[bib32] Grood ES , Suntay WJ. 1983. A joint coordinate system for the clinical description of three-dimensional motions: application to the knee. J Biomech Eng 105:136–44. 6865355 10.1115/1.3138397

[bib33] Grossnickle DM. 2017. The evolutionary origin of jaw yaw in mammals. Sci Rep 7:1–13. 28322334 10.1038/srep45094 PMC5359619

[bib34] Grossnickle DM 2020. Jaw roll and jaw yaw in early mammals. Nature 582:E6–8. 32555493 10.1038/s41586-020-2365-y

[bib35] Grossnickle DM , Weaver LN, Jäger KR, Schultz JA. 2022. The evolution of anteriorly directed molar occlusion in mammals. Zool J Linn Soc 194:349–65.

[bib36] Hautier L , Lebrun R, Cox PG. 2012. Patterns of covariation in the masticatory apparatus of hystricognathous rodents: implications for evolution and diversification. J Morphol 273:1319–37. 22833466 10.1002/jmor.20061

[bib37] Hautier L , Lebrun R, Saksiri S, Michaux J, Vianey-Liaud M, Marivaux L. 2011. Hystricognathy vs sciurognathy in the rodent jaw: a new morphometric assessment of hystricognathy applied to the living fossil Laonastes (Diatomyidae). PLoS One 6:e18698. 21490933 10.1371/journal.pone.0018698 PMC3072414

[bib38] Henderson SE , Desai R, Tashman S, Almarza AJ. 2014. Functional analysis of the rabbit temporomandibular joint using dynamic biplane imaging. J Biomech 47:1360–7. 24594064 10.1016/j.jbiomech.2014.01.051 PMC4010254

[bib39] Herring SW. 1976. The dynamics of mastication in pigs. Arch Oral Biol 21:473–80. 823928 10.1016/0003-9969(76)90105-9

[bib40] Herring SW. 1993. Functional morphology of mammalian mastication. Am Zool 33:289–99.

[bib41] Herring SW. 2003. TMJ anatomy and animal models. J Musculoskelet Neuronal Interact 3:391. 15758330 PMC2821032

[bib42] Herring SW , Decker JD, Liu Z-J, Ma T. 2002. Temporomandibular joint in miniature pigs: anatomy, cell replication, and relation to loading. Anat Rec 266:152–66. 11870598 10.1002/ar.10049

[bib43] Herring SW , Liu ZJ. 2001. Loading of the temporomandibular joint: anatomical and *in vivo* evidence from the bones. Cells Tissues Organs 169:193–200. 11455114 10.1159/000047882

[bib44] Herring SW , Scapino RP. 1973. Physiology of feeding in miniature pigs. J Morphol 141:427–60. 4760635 10.1002/jmor.1051410405

[bib45] Hiiemäe K. 1971a. The structure and function of the jaw muscles in the rat (Rattus norvegicus L.) II. Their fibre type and composition. Zool J Linn Soc 50:101–9.

[bib46] Hiiemäe K. 1971b. The structure and function of the jaw muscles in the rat (Rattus norvegicus L.) III. The mechanics of the muscles. Zool J Linn Soc 50:111–32.

[bib47] Hiiemäe K. 1984. Functional aspects of primate jaw morphology. In: Food acquisition and processing in primates. Boston, MA: Springer US. p. 257–81.

[bib48] Hiiemäe K , Houston WJB. 1971. The structure and function of the jaw muscles in the rat (*Rattus norvegicus* L.) I. Their anatomy and internal architecture. Zool J Linn Soc 50:75–99.

[bib49] Hiiemäe KM , Ardran GM. 1968. A cinefluorographic study of mandibular movement during feeding in the rat (Rattus norvegicus). J Zool 154:139–54.

[bib50] Hylander WL. 1979. The functional significance of primate mandibular form. J Morphol 160:223–39. 458862 10.1002/jmor.1051600208

[bib51] Hylander WL , Johnson KR. 1994. Jaw muscle function and wishboning of the mandible during mastication in macaques and baboons. Am J Phys Anthropol 94:523–47. 7977678 10.1002/ajpa.1330940407

[bib52] Hylander WL , Ravosa MJ, Ross CF, Wall CE, Johnson KR. 2000. Symphyseal fusion and jaw-adductor muscle force: an EMG study. Am J Phys Anthropol 112:469–92. 10918125 10.1002/1096-8644(200008)112:4<469::AID-AJPA5>3.0.CO;2-V

[bib53] Iriarte-Diaz J , Terhune CE, Taylor AB, Ross CF. 2017. Functional correlates of the position of the axis of rotation of the mandible during chewing in non-human primates. Zoology 124:106–18. 28993018 10.1016/j.zool.2017.08.006

[bib54] Kallen FC , Gans C. 1972. Mastication in the little brown bat, Myotis lucifugus. J Morphol 136:385–420. 5017451 10.1002/jmor.1051360402

[bib55] Kay RF , Hiiemäe KM. 1974. Jaw movement and tooth use in recent and fossil primates. Am J Phys Anthropol 40:227–56. 4815136 10.1002/ajpa.1330400210

[bib56] Knörlein BJ , Baier DB, Gatesy SM, Laurence-Chasen JD, Brainerd EL. 2016. Validation of XMALab software for marker-based XROMM. J Exp Biol 219:3701–11. 27655556 10.1242/jeb.145383

[bib57] Koolstra JH , Van Eijden TMGJ. 1995. Biomechanical analysis of jaw-closing movements. J Dent Res 74:1564–70. 7560417 10.1177/00220345950740091001

[bib58] Landry SO. 1954. The interrelationship of the New and Old World hystricomorph rodents. Berkeley, CA: University of California.

[bib59] Lauder GV. 1980. Evolution of the feeding mechanism in primitive actionopterygian fishes: a functional anatomical analysis of Polypterus, Lepisosteus, and Amia. J Morphol 163:283–317. 30170473 10.1002/jmor.1051630305

[bib60] Laurence-Chasen JD , Ramsay JB, Brainerd EL. 2019. Shearing overbite and asymmetrical jaw motions facilitate food breakdown in a freshwater stingray, *Potamotrygon motoro*. J Exp Biol 222:jeb197681. 31292213 10.1242/jeb.197681

[bib61] Lieberman DE , Crompton AW. 2000. Why fuse the mandibular symphysis? A comparative analysis. Am J Phys Anthropol 112:517–40. 10918127 10.1002/1096-8644(200008)112:4<517::AID-AJPA7>3.0.CO;2-4

[bib62] Luo Z-X , Crompton AW, Sun AL. 2001. A new mammaliaform from the early Jurassic and evolution of mammalian characteristics. Science 292:1535–40. 11375489 10.1126/science.1058476

[bib63] Luo ZX , Gatesy SM, Jenkins FA Jr, Amaral WW, Shubin NH. 2015. Mandibular and dental characteristics of Late Triassic mammaliaform Haramiyavia and their ramifications for basal mammal evolution. Proc Natl Acad Sci 112:E7101–9. 26630008 10.1073/pnas.1519387112 PMC4697399

[bib64] Luo Z-X , Meng QJ, Grossnickle DM, Liu D, Neander AI, Zhang YG, Ji Q. 2017. New evidence for mammaliaform ear evolution and feeding adaptation in a Jurassic ecosystem. Nature 548:326–9. 28792934 10.1038/nature23483

[bib65] Mao F , Hu Y, Li C, Wang Y, Chase MH, Smith AK, Meng J. 2020. Integrated hearing and chewing modules decoupled in a Cretaceous stem therian mammal. Science 367:305–8. 31806694 10.1126/science.aay9220

[bib66] Martin T , Jäger KR, Plogschties T, Schwermann AH, Brinkkötter JJ, Schultz JA, Koenigswald WV. 2020. Molar diversity and functional adaptations in Mesozoic mammals. Munich, Germany: Verlag Dr. Friedrich Pfeil.

[bib67] Menegaz RA , Baier DB, Metzger KA, Herring SW, Brainerd EL. 2015. XROMM analysis of tooth occlusion and temporomandibular joint kinematics during feeding in juvenile miniature pigs. J Exp Biol 218:2573–84. 26089531 10.1242/jeb.119438

[bib68] Mesnard M , Coutant JC, Aoun M, Morlier J, Cid M, Caix P. 2012. Relationships between geometry and kinematic characteristics in the temporomandibular joint. Comput Meth Biomech Biomed Eng 15:393–400. 10.1080/10255842.2010.539560 21264781

[bib69] Michaux J , Chevret P, Renaud S. 2007. Morphological diversity of Old World rats and mice (Rodentia, Muridae) mandible in relation with phylogeny and adaptation. J Zool Syst and Evol Res 45:263–79.

[bib70] Mills JRE. 1967. A comparison of lateral jaw movements in some mammals from wear facets on the teeth. Arch Oral Biol 12:645–61. 5228612 10.1016/0003-9969(67)90083-0

[bib71] Monteiro LR , Bonato V, Dos Reis SF. 2005. Evolutionary integration and morphological diversification in complex morphological structures: mandible shape divergence in spiny rats (Rodentia, Echimyidae). Evol Dev 7:429–39. 16174036 10.1111/j.1525-142X.2005.05047.x

[bib72] Montuelle SJ , Olson RA, Curtis H, Beery S, Williams SH. 2020. Effects of food properties on chewing in pigs: flexibility and stereotypy of jaw movements in a mammalian omnivore. PLoS One 15:e0228619. 32032365 10.1371/journal.pone.0228619 PMC7006907

[bib73] Moriuchi E , Hamanaka R, Koga Y, Fujishita A, Yoshimi T, Yasuda G, Kohara H, Yoshida N. 2019. Development and evaluation of a jaw-tracking system for mice: reconstruction of three-dimensional movement trajectories on an arbitrary point on the mandible. Biomed Eng Online 18. 10.1186/s12938-019-0672-z PMC6524240 31096969

[bib74] Offermans M , de Vree F. 1990. Mastication in springhares, *Pedetes capensis*: a cineradiographic study. J Morphol 205:353–67. 29865740 10.1002/jmor.1052050310

[bib75] Offermans M , de Vree F 1993. Electromyography and mechanics of mastication in the springhare, Pedetes capensis (Rodentia, Pedetidae). Belgian J Zool 123:231.

[bib76] Olson RA , Montuelle SJ, Chadwell BA, Curtis H, Williams SH. 2021. Jaw kinematics and tongue protraction–retraction during chewing and drinking in the pig. J Exp Biol 224:jeb239509. 33674496 10.1242/jeb.239509 PMC8077536

[bib77] Oron U , Crompton AW. 1985. A cineradiographic and electromyographic study of mastication in *Tenrec ecaudatus*. J Morphol 185:155–82. 4057264 10.1002/jmor.1051850203

[bib78] Orset E , Chaffanjon P, Bettega G. 2014. Temporomandibular joint model: anatomic and radiologic comparison between rat and human. Surg Radiol Anat 36:163–6. 23811934 10.1007/s00276-013-1159-4

[bib79] Osborn JW. 1989. The temporomandibular ligament and the articular eminence as constraints during jaw opening. J Oral Rehabil 16:323–33. 2795311 10.1111/j.1365-2842.1989.tb01348.x

[bib80] Patterson B. 1956. Early Cretaceous mammals and the evolution of mammalian molar teeth. Fieldiana Geol 13:1–105.

[bib81] Popowics TE , Herring SW. 2006. Teeth, jaws and muscles in mammalian mastication. In: Feeding in domestic vertebrates: from structure to behaviour. Wallingford: CABI. p. 61–83.

[bib82] Porto GG , Vasconcelos BCdE, Andrade ESdS, Silva-Junior VA. 2010. Comparison between human and rat TMJ: anatomic and histopathologic features. Acta Cirurgica Brasileira 25: 290–3. 20498943 10.1590/s0102-86502010000300012

[bib83] Qiao Y , Yi D, Reed DA, Mercuri LG, Chen D. 2022. A novel approach to establishing a temporomandibular joint fibrocartilage cell line. J Dent Sci 17:1378–86. 35784155 10.1016/j.jds.2022.04.017 PMC9236962

[bib84] Ravosa MJ , Hogue AS. 2004. Function and fusion of the mandibular symphysis in mammals: a comparative and experimental perspective. In: Anvthropoid origins: new visions. New York, NY: Springer. p. 413–62.

[bib85] Reed DA , Yotsuya M, Gubareva P, Toth PT, Bertagna A. 2019. Two-photon fluorescence and second harmonic generation characterization of extracellular matrix remodeling in post-injury murine temporomandibular joint osteoarthritis. PLoS One 14:e0214072. 30897138 10.1371/journal.pone.0214072 PMC6428409

[bib86] Reed DA , Zhao Y, Han M, Mercuri LG, Miloro M. 2021. Mechanical loading disrupts focal adhesion kinase activation in mandibular fibrochondrocytes during murine temporomandibular joint osteoarthritis. J Oral Maxillofac Surg 79:2058–e1. 10.1016/j.joms.2021.05.001 PMC8500914 34153254

[bib87] Reilly SM , McBrayer LD, White TD. 2001. Prey processing in amniotes: biomechanical and behavioral patterns of food reduction. Comp Biochem Physiol A: Mol Integr Physiol 128:397–415. 11246036 10.1016/s1095-6433(00)00326-3

[bib88] Ross CF , Eckhardt A, Herrel A, Hylander WL, Metzger KA, Schaerlaeken V, Washington RL, Williams SH 2007. Modulation of intra-oral processing in mammals and lepidosaurs. Integr Comp Biol 47:118–36. 21672825 10.1093/icb/icm044

[bib89] Ross CF , Iriarte-Diaz J. 2014. What does feeding system morphology tell us about feeding?. Evol Anthropol: Issues, News, Rev 23:105–20. 10.1002/evan.21410 24954218

[bib90] Ross CF , Iriarte-Diaz J. 2019. Evolution, constraint, and optimality in primate feeding systems. In: Feeding in vertebrates. Cham: Springer. p. 787–829.

[bib91] Roveroni RC , Parada CA, Cecılia M, Veiga FA, Tambeli CH. 2001. Development of a behavioral model of TMJ pain in rats: the TMJ formalin test. Pain 94:185–91. 11690732 10.1016/S0304-3959(01)00357-8

[bib92] Sangani D , Suzuki A, VonVille H, Hixson JE, Iwata J. 2015. Gene mutations associated with temporomandibular joint disorders: a systematic review. OAlib 2:e1583. 27695703 10.4236/oalib.1101583 PMC5045035

[bib93] Scapino R. 1981. Morphological investigation into functions of the jaw symphysis in carnivorans. J Morphol 167:339–75. 7241602 10.1002/jmor.1051670308

[bib94] Schultz JA. 2020. Eat and listen—how chewing and hearing evolved. Science 367:244–6. 31949065 10.1126/science.aba3808

[bib95] Schultz JA , Krause DW, Von Koenigswald W, Dumont ER. 2014. Dental function and diet of Vintana sertichi (Mammalia, Gondwanatheria) from the Late Cretaceous of Madagascar. J Vertebr Paleontol 34:182–202.

[bib96] Schwarz D , Konow N, Roba YT, Heiss E. 2020. A salamander that chews using complex, three-dimensional mandible movements. J Exp Biol 223:jeb220749. 31988164 10.1242/jeb.220749

[bib98] Simpson JJ. 1936. Studies of the earliest mammalian. Dental Cosmos 78:791–800.

[bib99] Sperry MM , Kartha S, Winkelstein BA, Granquist EJ. 2019. Experimental methods to inform diagnostic approaches for painful TMJ osteoarthritis. J Dent Res 98: 388–97. 30819041 10.1177/0022034519828731 PMC6429670

[bib100] Stilson KT. 2021. The Kinematics and Neurofeedback of Mastication in Didelphis virginiana. [PhD Thesis]. [Chicago (IL)]: University of Chicago.

[bib101] Stilson KT , Luo Z-X, Li P, Olson S, Ross CF. 2023 Three-dimensional mandibular kinematics of mastication in the marsupial *Didelphis virginiana*. Philos Trans R Soc B 378:20220548. 10.1098/rstb.2022.0548 PMC10577026 37839456

[bib102] Stover KK , Sidote J, Williams SH. 2017. An ontogenetic perspective on symphyseal fusion, occlusion and mandibular loading in alpacas (Vicugna pacos). Zoology 124:95–105. 28811168 10.1016/j.zool.2017.06.006

[bib103] Sun JL , Yan JF, Yu SB, Zhao J, Lin QQ, Jiao K. 2020. MicroRNA-29b promotes subchondral bone loss in TMJ osteoarthritis. J Dent Res 99:1469–77. 32693649 10.1177/0022034520937617

[bib104] Takeda M , Tanimoto T, Ikeda M, Nasu M, Kadoi J, Yoshida S, Matsumoto S. 2006. Enhanced excitability of rat trigeminal root ganglion neurons via decrease in A-type potassium currents following temporomandibular joint inflammation. Neuroscience 138:621–30. 16387448 10.1016/j.neuroscience.2005.11.024

[bib105] Teaford MF , Byrd KE. 1989. Differences in tooth wear as an indicator of changes in jaw movement in the guinea pig *Cavia porcellus*. Arch Oral Biol 34:929–36. 2610627 10.1016/0003-9969(89)90048-4

[bib106] Tuijt M , Koolstra JH, Lobbezoo F, Naeije M. 2010. Differences in loading of the temporomandibular joint during opening and closing of the jaw. J Biomech 43:1048–54. 20096414 10.1016/j.jbiomech.2009.12.013

[bib107] Ungar PS , Sues HD. 2019. Tetrapod teeth: diversity, evolution, and function. In: Feeding in Vertebrates. Cham: Springer. p. 385–429.

[bib108] Walker WF , Homberger DG. 1997. Anatomy and dissection of the rat. New York, NY: Macmillan.

[bib109] Wall CE. 1999. A model of temporomandibular joint function in anthropoid primates based on condylar movements during mastication. Am J Phys Anthropol: The Official Publication of the Am Assoc Phys Anthropologists 109:67–88. 10.1002/(SICI)1096-8644(199905)109:1<67::AID-AJPA7>3.0.CO;2-F 10342466

[bib110] Weijs WA. 1975. Mandibular movements of the albino rat during feeding. J Morphol 145:107–24. 1111422 10.1002/jmor.1051450107

[bib111] Weijs WA , Dantuma R. 1975. Electromyography and mechanics of mastication in the albino rat. J Morphol 146:1–33. 1171253 10.1002/jmor.1051460102

[bib112] Williams SH. 2019. Feeding in Mammals: comparative, Experimental, and Evolutionary Insights on Form and Function. In: Feeding in vertebrates. Cham: Springer,. p. 695–742.

[bib113] Williams SH , Stover KK, Davis JS, Montuelle SJ. 2011. Mandibular corpus bone strains during mastication in goats (Capra hircus): a comparison of ingestive and rumination chewing. Arch Oral Biol 56:960–71. 10.1016/j.archoralbio.2011.02.014 21411059

[bib114] Williams SH , Vinyard CJ, Wall CE, Hylander WL. 2007. Masticatory motor patterns in ungulates: a quantitative assessment of jaw muscle coordination in goats, alpacas and horses. J Exp Zool A: Ecol Genet Physiol 307:226–40. 17436331 10.1002/jez.362

[bib115] Williams SH , Wall CE, Vinyard CJ, Hylander WL. 2008. Symphyseal fusion in selenodont artiodactyls: new insights from∼ *in vivo* and comparative data. In: Primate craniofacial function and biology. Boston, MA: Springer US. p. 39–61.

[bib116] Yatabe M , Zwijnenburg A, Megens CCEJ, Naeije M. 1997. Movements of the mandibular condyle kinematic center during jaw opening and closing. J Dent Res 76: 714–9. 9062566 10.1177/00220345970760021301

[bib117] Yotsuya M , Bertagna AE, Hasan N, Bicknell S, Sato T, Reed DA. 2019. Neuron/glial antigen 2-type vi collagen interactions during murine temporomandibular joint osteoarthritis. Sci Rep 9:1–10. 30635602 10.1038/s41598-018-37028-1 PMC6329769

[bib118] Yotsuya M , Iriarte-Diaz J, Reed DA. 2020. Temporomandibular joint hypofunction secondary to unilateral partial discectomy attenuates degeneration in murine mandibular condylar cartilage. Bull Tokyo Dent Coll 61:9–19. 32101827 10.2209/tdcpublication.2019-0008 PMC8304519

[bib119] Zazhigin VS , Voyta LL. 2019. Northern Asian Pliocene–Pleistocene beremendiin shrews (Mammalia, Lipotyphla, Soricidae): a description of material from Russia (Siberia), Kazakhstan, and Mongolia and the paleobiology of Beremendia. J Paleontol 93:1234–57.

